# The Promise of Molecular and Genomic Techniques for Biodiversity Research and DNA Barcoding of the Arabian Peninsula Flora

**DOI:** 10.3389/fpls.2018.01929

**Published:** 2019-01-21

**Authors:** Kareem A. Mosa, Sanjay Gairola, Rahul Jamdade, Ali El-Keblawy, Khawla Ibrahim Al Shaer, Eman Khalid Al Harthi, Hatem A. Shabana, Tamer Mahmoud

**Affiliations:** ^1^Department of Applied Biology, College of Sciences, University of Sharjah, Sharjah, United Arab Emirates; ^2^Department of Biotechnology, Faculty of Agriculture, Al-Azhar University, Cairo, Egypt; ^3^Sharjah Seed Bank and Herbarium, Sharjah Research Academy, Sharjah, United Arab Emirates; ^4^Plant Biotechnology Laboratory, Sharjah Research Academy, Sharjah, United Arab Emirates

**Keywords:** Arabian Peninsula, molecular markers, genomic techniques, plant biodiversity, DNA barcoding, herbarium collections

## Abstract

The Arabian Peninsula is known to have a comprehensive and rich endowment of unique and genetically diverse plant genetic resources. Analysis and conservation of biological diversity is a crucial issue to the whole Arabian Peninsula. The rapid and accurate delimitation and identification of a species is crucial to genetic diversity analysis and the first critical step in the assessment of distribution, population abundance and threats related to a particular target species. During the last two decades, classical strategies of evaluating genetic variability, such as morphology and physiology, have been greatly complemented by phylogenetic, taxonomic, genetic diversity and breeding research molecular studies. At present, initiatives are taking place around the world to generate DNA barcode libraries for vascular plant flora and to make these data available in order to better understand, conserve and utilize biodiversity. The number of herbarium collection-based plant evolutionary genetics and genomics studies being conducted has been increasing worldwide. The herbaria provide a rich resource of already preserved and identified material, and these as well as freshly collected samples from the wild can be used for creating a reference DNA barcode library for the vascular plant flora of a region. This review discusses the main molecular and genomic techniques used in plant identification and biodiversity analysis. Hence, we highlight studies emphasizing various molecular techniques undertaken during the last 10 years to study the plant biodiversity of the Arabian Peninsula. Special emphasis on the role of DNA barcoding as a powerful tool for plant biodiversity analysis is provided, along with the crucial role of herbaria in creating a DNA barcode library.

## Introduction

**FIGURE 1:**
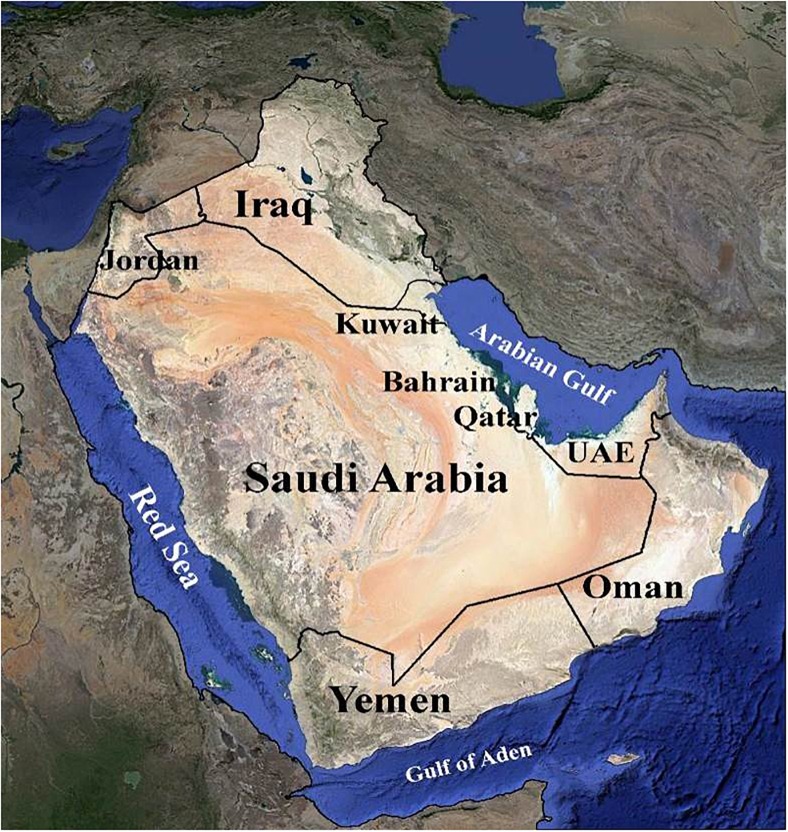
Map Arabian Peninsula representing nine countries.

The Arabian Peninsula is a topographically diverse region ranging from rainless vast sandy and rock deserts such as the Rub’ al-Khali and the Great Nafud, to mist-covered mountains reaching over 3,000 m in height along the escarpment of the southwest (Ghazanfar and Fisher, 1998; Al Farhan et al., 2008). It has arid to hyper-arid climates (Loughland and Cunningham, 2002) (Figure 1). Such topographic heterogeneity has given rise to a diversity of habitats and a correspondingly diverse array of both plant and animal species. The vegetated landscape of the Peninsula includes woodland, bushland, thicket, shrubland, scrub, mangrove, and desert with seasonal annuals. The species composition shows a mix of Holarctic and Palaeotropical elements (Miller and Cope, 1996; Knees et al., 2010). The Arabian Peninsula is known to have a rich endowment of unique and genetically diverse plant genetic resources. According to Miller and Morris (2004), the Peninsula is home to approximately 3,418 plant species, out of which roughly 357 (11.5%) are endemic. Hence, the flora of the Arabian Peninsula has great ecological and socio-economic importance (El-Keblawy, 2018). The United Arab Emirates (UAE) has about 830 plant species. However, detailed studies of the UAE flora and plant communities are scarce, with data often lacking (Gairola et al., 2017).

The natural ecosystems of the Peninsula, like many other arid regions, are susceptible to habitat fragmentation. The major threats to plant diversity include habitat loss and degradation, overgrazing by livestock, overexploitation, off-road driving, pollution and anthropogenic climate change (Ferguson et al., 1998; Ghazanfar and Fisher, 1998; Abu-Zinada et al., 2008; Al Farhan et al., 2008; Brown et al., 2008; El-Keblawy et al., 2009, 2015; Ghazanfar, 2010; El-Keblawy, 2017, 2018). High mountains harbor a unique and large portion of the Arabian Peninsula’s biodiversity. Mountain flora are often endemic as many species remain isolated at high elevations (Thomas, 2010). For example, the Hajar Mountains in eastern Arabia (Northern Oman and the eastern UAE) are among the highest mountains in the Arabian Peninsula and have been classified as a local center of endangered and endemic plants in the eastern Arabia (Miller and Nyberg, 1990; Patzelt, 2008). A total of 75 species are locally endemic to the high mountains of Ru’us al-Jibal (Feulner, 2011; El-Keblawy, 2014). These biodiversity hotspots would be seriously affected by expected global warming, and consequently their endemic and endangered species would be under threat (El-Keblawy, 2014). This emphasizes the threat and distinctiveness of the flora in the high mountains and, consequently, the importance of their conservation.

Among the main causes of native plant extinction is the introduction of invasive species (Barbier et al., 2013). Biological invasions are recognized as one of the most important causes of ecosystem degradation as well as community structure, local species and biodiversity losses worldwide (Pyšek et al., 2012). In the Arabian Peninsula, *Prosopis juliflora* has been introduced for the rehabilitation of degraded afforestation lands (El-Keblawy and Abdelfatah, 2014). It has invaded many habitats and significantly reduced the native floral diversity (El-Keblawy and Abdelfatah, 2014; El-Keblawy et al., 2015). As it forms impenetrable shrubby thickets, *P. juliflora* has a very negative impact on the native flora as it depletes the water-table, thus indirectly starving native plants of other species due to lack of moisture and nutrients (El-Keblawy and Ksiksi, 2005; El-Keblawy et al., 2015). In addition, this species produces allelochemicals that kill understory native plants (El-Keblawy and Abdelfatah, 2014).

Conservation of individual species and genetically biodiverse hot spots require the use of recent molecular techniques for proper species identification. Additionally, assessing the genetic relationship between native plants and their close relatives (among domesticated crops) would help in defining and inserting these genes.

The valuable biological resources of the Peninsula are an integral part of the natural heritage and provide important ecosystem-related goods and services. Therefore, there is a need to conserve and manage these species. Recently, interest in biodiversity studies has increased through the use of promising approaches like molecular and morphological data for identifying taxa, studies of species complexes, and in aiding species delimitation and identification.

The rapid and accurate delimitation and identification of a species is the basis for biodiversity conservation and is one of the keys to improving species management and conservation (Trias-Blasi and Vorontsova, 2015). Molecular and genomics techniques are now being utilized in a variety of plant biodiversity studies, including identification of plant species (e.g., Savolainen and Karhu, 2000), creating DNA barcodes for large taxonomic groups (e.g., Ojeda et al., 2014), addressing discrete taxonomic problems (e.g., Feau et al., 2011), discovery of new taxa (e.g., Bell et al., 2012), species conservation tools (e.g., Yesson et al., 2011), and building phylogenetic trees, enabling studies on community ecology (e.g., Joly et al., 2013; Kress et al., 2015). DNA barcoding has become increasingly common since it was proposed in 2003. The Consortium for the Barcode of Life (CBOL Plant Working Group et al., 2009) plant working group recommended the 2-loci combination of *rbcL* and *matK* as the standard chloroplast DNA barcode for land plants. However, there have been persistent calls from some researchers (Parmentier et al., 2013; Li et al., 2015; Daru et al., 2016) to include *ITS* into the core barcodes as well. In addition, Chen et al. (2010) proposed that the ITS2 region could potentially be used as a standard DNA barcode, especially for identifying medicinal plants and their closely related species.

From the botanical perspective, herbarium specimens are an important source of DNA for plant research. Recently, Heberling and Isaac (2017) suggested that herbarium specimens can be viewed as “exaptations,” as the current use of collections reaches far beyond their originally anticipated uses. Researchers have documented that DNA is often preserved in herbarium samples, allowing amplification and successful sequencing. However, the extraction of plant material from herbarium specimens suitable for DNA analysis has been investigated with varying degrees of success depending on the technique used, age of material, preservation methods, and the conditions in which specimens have been stored (e.g., Rogers and Bendich, 1985; Liston et al., 1990; Lister et al., 2008; Andreasen et al., 2009; Särkinen et al., 2012; Kuzmina et al., 2017). The recent progress achieved in barcoding preserved specimens in herbaria, museums and other repositories increases the value of these collections as a source of genetic diversity information that is relevant to ecology, population genetics, and evolutionary and conservation biology (Wandeler et al., 2007).

Recently, some attention has been devoted to genetic diversity analysis, species delimitation and barcoding desert plants in the UAE (Gairola et al., 2018). Enan et al. (2017) highlighted that DNA barcoding has demonstrated promising outcomes from both fresh and herbarium samples of desert plants in the UAE. The authors concluded that the *rbcL* regions demonstrated a realistic potential to distinguish the UAE species under investigation into the appropriate family and genus. However, the molecular identification and phylogenetic analysis of the UAE’s flora is yet to be investigated. The combination of *rbcL* and *matK* has been successful in barcoding some of the desert plants in the UAE (Enan et al., 2017). Recently, a project to barcode the entirety of UAE flora has been initiated at the Sharjah Research Academy (SRA). Conservation of biological diversity is a crucial issue for the whole Arabian Peninsula and it is vital that DNA barcoding of the regional flora is attempted.

In this article, we discuss the progress that has been made in using molecular and genomic techniques for biodiversity studies in the Arabian Peninsula, accompanied by studies highlighting the particular forms of molecular techniques undertaken in the Arabian Peninsula during the last 10 years. Particular emphasis is given to the potential of DNA barcoding as a powerful tool for molecular identification as well as in various taxonomic, molecular ecology, phylogenetics and biodiversity research. Additionally, we have devoted more attention to the crucial role of herbaria in creating a reference DNA barcode library for rapid and accurate identification of floral diversity.

## Standard Molecular and Genomic Techniques in Plant Biodiversity Analysis

Molecular markers, specifically DNA-based markers, can provide a good estimation of genetic diversity. A molecular marker is a DNA sequence with a known location on the chromosome and whose inheritance can be monitored. The development of molecular markers depends on polymorphisms found in DNA, and the information obtained can be used to measure the relationships between organisms and other genetic diversity studies (Hoshino et al., 2012).

There are many molecular markers available today for researchers in the plant sciences field. Although some can be identical, similar or synonymous, many differ in their principles, methodologies, applications, resolving power to detect genetic differences, and in the type of data they generate. Each molecular technique has its own advantages and disadvantages, but the majority of these techniques can be used for several applications like genetic mapping, marker assisted selection and phylogenic analysis (Semagn et al., 2006; Arif et al., 2010a; Mishra et al., 2014). In order for a molecular marker to be considered as ideal for usage in a specific technique, it should possess certain features, including genomic abundance and high level of polymorphism, easy and fast assay, co-dominance of alleles and high reproducibility (Mondini et al., 2009). Obtaining a single molecular marker that presents all the mentioned features among the various types is difficult. However, depending on the type of study and the technique to be used, a suitable marker system that satisfies the requirements can be chosen (Spooner et al., 2005).

**FIGURE 2:**
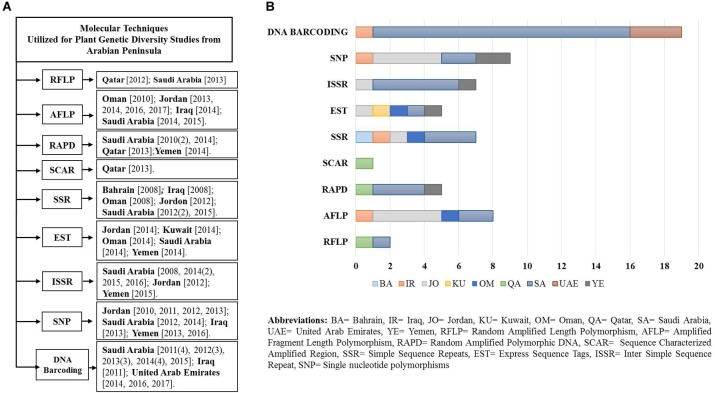
**(A)** Flow diagram of the studies (peer reviewed) undertaken in a decade using various molecular markers and genomics techniques from the Arabian Peninsula. **(B)** Plot of studies utilizing various molecular techniques.

To evaluate relevant studies undertaken in the Arabian Peninsula during the last 10 years on plant genetic diversity using various molecular and genomic techniques, NCBI PubMed was used on the 15th of June 2017. We obtained relevant search results through multiple queries using the advance search builder [Query: ((plants) AND <marker>) AND <country>]. Further results were filtered to restrict the search criteria to one decade (2007–2017). Some articles that were not retrieved through the advance search query were added from their citations in the retrieved articles by reviewing them critically. Obtained results were segregated according to the country and molecular marker, and a flow chart with bar graph was generated (Figures 2A,B). Discussed below are the long-established molecular markers used to study plant biodiversity in the Arabian Peninsula.

### Restriction Fragment Length Polymorphism (RFLP)

Restriction fragment length polymorphism (RFLP) analysis is a technique used to differentiate among species by analyzing the characteristic pattern formed by the digested DNA fragments. The RFLP markers tend to be inherited as simple Mendelian co-dominant alleles (Agarwal et al., 2008), as both alleles in a heterozygous sample will be detected. However, their DNA rearrangements are due to evolutionary process, point mutations within the restriction enzyme recognition site or unequal crossing over (Kumar et al., 2009; Mishra et al., 2014). The resultant patterns from different samples, formed by separation of bands using agarose or polyacrylamide gel electrophoresis, are analyzed to differentiate among the plant species from which the samples are to be taken (Agarwal et al., 2008). These different patterns formed by digesting DNA obtained from samples of different plant species are due to polymorphisms or changes in the recognition sites.

There are few studies reporting the utilization of RFLP markers for plant biodiversity analysis in the Arabian Peninsula (Figure 2). A traditional approach for detecting polymorphism from Saudi Arabian fenugreek using RFLP markers was adopted by Haliem and Al-Huqail (2013). They used four RFLP restriction enzymes that resulted in higher polymorphism (about 88.7%) in comparison to when the enzymes were absent during SDS-PAGE (only 80%). Additionally, Al-Mahmoud et al. (2012) used RFLP markers for sex differentiation in the date palm (*Phoenix dactylifera*) at the earliest stages. The primers designed for this study were gender-specific, which revealed higher accuracy (90%) in distinguishing date palm gender across multiple varieties.

### Amplified Fragment Length Polymorphism (AFLP)

Amplified fragment length polymorphism (AFLP) is a highly sensitive method which combines both RFLP and PCR technologies. DNA of any origin, complexity, or even partially degraded DNA can be analyzed using this method. The concept of AFLP includes the amplification of digested fragments of the genomic DNA by PCR (Vos et al., 1995; Mueller and Wolfenbarger, 1999). This technique is capable of ‘genome representation,’ which means that all representative DNA regions that are distributed randomly around the genome will be screened at the same time (Meudt and Clarke, 2007; Idrees and Irshad, 2014). The polymorphisms are detected as differences in the length of the amplified fragments and are considered as dominant markers (Sunnucks, 2000; Belaj et al., 2003; Campbell et al., 2003; Schlötterer, 2004; Kumar et al., 2009). However, they can also act as co-dominant markers at certain loci (Mishra et al., 2014).

Several studies reported the observance of genetic diversity and phylogenetic relationships in the Arabian Peninsula (Figure 2). For example, from the southern region of Oman, the genetic variation in local banana cultivars (*Musa cvs.*) was studied using AFLP. They successfully identified about 92.5% of polymorphism and recommended AFLP as an efficient marker in determining the relationship between cultivars (Opara et al., 2010). Another study in Jordan reported higher variability in the genetic structure of *Stipa* populations (*n* = 120) in semi-desert species than in ruderal habitats (Hamasha et al., 2013). The authors attributed the genetic variation to a steep climatic gradient that may have shaped the genetic structure of plant populations. El Rabey et al. (2014) indicated that this technique is also able to successfully recognize different genotypes in plant species by analyzing their phylogenetic relationship. These authors analyzed 10 species from the genus *Hordeum* collected across Jordan and Iraq using AFLP markers. Their study clustered the taxa according to their genotypes into two main groups representing the Old and New World taxa. Additionally, Alghamdi et al. (2014) assessed the genetic diversity among 35 introduced lentil genotypes (*Lens culinaris*) from Saudi Arabia and reported a 100% detection rate of polymorphism. Similarly, development of DNA fingerprints that may enable species discrimination using AFLP molecular markers was reported in 10 date palm (*Phoenix dactylifera*) cultivars from Saudi Arabia; the polymorphism levels ranged from 63% to 84%, with an average of 76%. Furthermore, Ammar et al. (2015) explored the molecular diversity among 40 faba beans (*Vicia faba*) using AFLP markers and reported considerable genetic diversity in the studied genotypes. Another study reported the heterogeneity among the *Salvia spinosa* and *Salvia syriaca* populations from different phytogeographical regions of Jordan, indicating an increase in genetic diversity in relation with increased temperatures. In this study, populations’ heterogeneity increased from moist to arid environments, which could be the driving factor behind the observed variation and genetic diversity (Al-Gharaibeh et al., 2016, 2017). From these studies, it is clear that AFLP marker systems possess a high level of resolution and discrimination power, and hence can be used in varietal discrimination of species.

### Random Amplified Polymorphic DNA (RAPD)

Random amplified polymorphic DNA (RAPD) is a PCR-based technique for identifying genetic variation. The RAPD technique is efficient in screening and detecting polymorphisms at several discrete DNA loci (Kumari and Thakur, 2014). RAPD is considered as a genetic marker since it is dominant and randomly distributed. RAPD could be performed at different levels; ranging from the individual to the species level. Additionally, RAPD is used to estimate the genetic diversity of plant populations in variability analysis (Ndoye-Ndir et al., 2008) and during genotyping to determine diversity and relatedness (Akbulut et al., 2009).

Random amplified polymorphic DNA markers were used to successfully characterize and identify 11 Saudi Arabian plant species of desert origin, some with known medicinal value (Arif et al., 2010b). Since these plant species hold significant value, the study was conducted to help protect such species and allow for their appropriate use. The RAPD markers produced distinct banding patterns, hence allowing successful species discrimination (Arif et al., 2010b). Similarly, Sheeja et al. (2013) used RAPD markers for the discrimination of *Knema andamanica* from neighboring taxa in Qatar. The authors used clustering analysis to separate *K. andamanica* from other closely related taxa. RAPD has also been used to explore genetic diversity among populations. In a study from Saudi Arabia, Hammad and Qari (2010) revealed genetic variation within and between 12 populations of *Zygophyllum* species (*Z. coccineum*, *Z. album* and *Z. aegyptium*). However, *Z. coccineum* demonstrated higher levels of genetic variation than the other species (Hammad and Qari, 2010). Similarly, Haliem and Al-Huqail (2014) studied the genetic variation in fenugreek (*Trigonella foenum-graecum*) (*n* = 7) from Saudi Arabia and Yemen using RAPD, concluding that this species exhibited a high level of polymorphism (94.2%).

### Sequence Characterized Amplified Region (SCAR)

Sequence characterized amplified region (SCAR) is a polymorphic DNA fragment of a known sequence. SCAR markers are reliable and reproducible and thus well suited for many applications (Yuskianti and Shiraishi, 2010). The SCAR assay is a PCR-based assay which involves the identification of a DNA fragment by PCR amplification using a pair of specific oligonucleotide (15–30 bp) primers that are designed from nucleotide sequences of cloned RAPD (or other markers) fragments related to the trait of interest (Bhagyawant, 2016).

Based on our literature analysis, we were able to find only one collaborative study between India and Qatar reporting molecular identification of *Knema andamanica* by using SCAR markers (Figure 2) that were developed by cloning RAPD markers. This technique successfully identified *K. andamanica* from the most genetically related species. The authors referred to the SCAR technique as a robust methodology and suggested its use as a DNA barcode marker in species authentication (Sheeja et al., 2013).

### Microsatellites and Minisatellites

Polymorphic loci of DNA consisting of short repeat motifs (1–6 base pairs) are called microsatellites. Simple Sequence Repeats (SSRs), Simple Sequence Length Polymorphism (SSLP), Sequence Tagged Microsatellite Sites (STMSs) and Short Tandem Repeats (STRs) are other names of the smallest class of repetitive sequences in DNA, which are the microsatellites. Microsatellites can be found in both the transcribed and non-transcribed regions of the genome. Their abundance and polymorphism make them particularly valuable for describing variation among individuals and populations (Westman and Kresovich, 1997; Varshney et al., 2005).

Minisatellites are also known as variable of tandem repeats loci, or VNTR loci, because they are part of the DNA and consist of short base pairs (10–60) that are repeated and widely distributed in the plants’ genome. These loci can be used as molecular markers because their repeats vary in number among individuals and populations. In plants, minisatellites are common and have been used as markers to assess variation between and within species (Westman and Kresovich, 1997). Multilocus profiles produced by using minisatellites as molecular markers are unique when compared between individuals. This is due to the fact that minisatellites have a high mutation rate, hence the level of polymorphism among individuals is also very high (Agrawal and Shrivastava, 2014).

Several studies have used SSR markers on various plant species to report genetic diversity, sex differentiation, determination of population structure and polymorphism throughout the Arabian Peninsula (Figure 2). Among those, the date palm (*Phoenix dactylifera*) was one of the most extensively studied taxa. In Oman, Bahrain and Iraq, the date palm was considered for its genetic diversity among clonal genotypes by Al-Ruqaishi et al. (2008) using microsatellite markers (*n* = 10). Their analysis revealed identical genetic fingerprints. In Saudi Arabia, Elmeer and Mattat (2012) studied sex differentiation in date palms (*Phoenix dactylifera*) and detected 254 microsatellite loci; of these, 22 loci could be used to identify nine out of 12 male date palm samples (75%). In addition, Elmeer and Mattat (2015) investigated the genetic diversity in the date palm germplasm (female date palms cultivars). Their results indicated that all of the cultivars displayed different levels of dissimilarity, but they were still grouped together. These studies successfully demonstrated the use of SSR alleles to distinguish between large numbers of date palm cultivars. Alatar et al. (2012) detected polymorphism in different medicinal plant species in Saudi Arabia and provided a protocol for DNA isolation and the SSR-PCR method. A microsatellite-based investigation of gene flow between wild barley *Hordeum spontaneum* and cultivated barley *Hordeum vulgare* was also performed by Hubner et al. (2012) in Jordan. The study indicated that the SSR marker related the genetic population structure to major ecogeographic regions of Jordan. However, no single study utilizing minisatellites in the Arabian Peninsula has been reported yet; this may be due to their lack of potential in discriminating species due to hypervariability.

### Expressed Sequence Tag (EST)

Expressed sequence tags (ESTs) are portions of expressed genes (50–400 bp) from a cDNA clone that corresponds to an mRNA. For EST to be used as a molecular marker, cDNA libraries must first be constructed (Semagn et al., 2006; Idrees and Irshad, 2014).

Expressed sequence tags are developed and publicly available for most crop plants. The EST databases are a valuable resource for the development of molecular markers that could be used in evolutionary studies or to identify gene transcripts that are important in gene discovery. There is an increased generation of ESTs with advancement in high throughput functional genomics via approaches as Serial Analysis of Gene Expression (SAGE). Such information could provide an insight on sequence determination, gene expression and regulation, and for developing highly valuable molecular markers such as EST-based RFLPs, SSRs, single nucleotide polymorphism (SNPS) and Cleaved Amplified Polymorphic Sequences (CAPS) (Semagn et al., 2006; Sedláček et al., 2010; Idrees and Irshad, 2014). Since EST-derived markers come from mRNA (a transcribed region of genome), they are likely to be conserved across a broader taxonomic range than other types of markers (Pashley, 2006). EST databases are sources of SSRs or microsatellites that can be developed as ortholog-specific EST-SSR markers and used in applications related to the genotypes of many plant species (Ellis and Burke, 2007). Pattern-finding programs can be used to identify SSRs in ESTs. Such markers have been reported in various plant species, including *Arabidopsis thaliana*, cacao, and sugarcane (Seong et al., 2015). EST-SSRs have a higher probability of being functionally associated with differences in gene expression than genomic SSRs. An advantage of EST-SSR is being more transferable across closely related genera compared with unknown SSRs in introns or UTRS (Varshney et al., 2005). Hence, EST-SSRs are well-suited for studying polymorphisms and genetic diversity as they are easier to comprehend (Seong et al., 2015). With the advent of technology, EST is being used for developing transcriptome research. It has been widely applied in the analysis of gene expression and gene function in various love spectrums (Adams et al., 1991; Boguski et al., 1994).

Plant biodiversity studies on ESTs have not received much attention in the Arabian Peninsula; Miryeganeh et al. (2014) studied the level of inter-population migration of long-distance seed dispersal in *Ipomoea pes-caprae* using an EST marker sampled from Oman, Jordan, Kuwait, Saudi Arabia, and Yemen. They concluded that the migration of seeds by sea drift was enough to connect these distant populations.

### Inter Simple Sequence Repeat (ISSR)

A DNA segment (below 100–300 bp) positioned in between two identical microsatellite repeat regions (usually 16–25 bp long) oriented in the opposite direction is termed Inter Simple Sequence Repeat (ISSR) (Zietkiewicz et al., 1994; Culley and Wolfe, 2001; Reddy et al., 2002). ISSR fingerprinting has the advantage of not requiring sequence knowledge for primer construction as well as demonstrating specificity of SSR markers. ISSR markers are randomly distributed throughout the genome and exhibit high polymorphism with different degrees.

The application of the ISSR markers has been demonstrated extensively throughout the Arabian Peninsula (Figure 2). For example, Obeed et al. (2008) used ISSR to investigate fruit properties and the genetic diversity of five ber (*Ziziphus mauritiana*) cultivars (Komethry, Pakstany, Um-sulaem, Toffahy, and Peyuan) grown in Saudi Arabia. Moreover, ISSR was used for the molecular characterization and fingerprint identification of the ber cultivars. The authors were able to uniquely characterize and differentiate between the five ber genotypes successfully. In another study from Jordan, the genetic stability of micropropagated *Moringa peregrina* plants was assessed with ISSR markers. No polymorphism was observed, indicating the genetic integrity of *in vitro* propagated plants (Al Khateeb et al., 2012).

More recently, a study from Saudi Arabia revealed the development of DNA fingerprints, early sex identification and the aiding of conservation when using the ISSR marker (Sabir et al., 2014a). These authors developed DNA fingerprints using ISSR molecular markers to characterize 10 date palm (*Phoenix dactylifera*) cultivars as well as to estimate the genetic diversity amongst them. Their ISSR results indicated that the levels of polymorphism ranged from 20 to 100% among cultivars, with an average of 85% (Sabir et al., 2014a). Gaafar et al. (2014) evaluated the genetic variations across populations and geographical regions of the endangered *Breonadia salicina* (Rubiaceae). The authors used ISSR markers and revealed that the genetic diversity levels were low within some populations, but relatively high amongst others. Another study suggested the use of ISSR markers to detect phenotypic variation amongst 15 genotypes of *Sorghum* landrace grown in Saudi Arabia and Yemen. In this case, eight genotypes of *Sorghum bicolor* were successfully differentiated into two clusters, one with dark grains and the other with white grains (Basahi, 2015). Recently, Al-Ameri et al. (2016) used ISSR markers for the early sex determination of date palms in Saudi Arabia. They were able to identify sex at the seedling stage, where it is economically important to avoid males more than the need of a farm. The ISSR primers used in the above studies are useful to provide reproducible results for genetic discrimination, sex identification, plant propagation and commercial cultivation. El Rabey et al. (2015) analyzed 14 cereal germplasms belonging to five cereal species (rice, wheat, barley, sorghum, and maize) using polymerase chain reaction (PCR). Here, 10 ISSR primers and 15 random RAPD primers were utilized as genetic markers in order to analyze the phylogenetic relationships between their genomes. As a result, 109 ISSR markers between 400–3,000 bp and 130 RAPD markers were scored within the same cultivars. The numerical taxonomy system of the multivariate statistical (NTSYS-pc) program arranged the samples into two clusters. The first cluster included closely related maize and sorghum cultivars. On the other hand, the second cluster included rice, wheat and barley in which the two latter cultivars specifically appeared to be closer to each other than to rice. The outcome concludes that both ISSR and RAPD markers can be implemented successfully to study the genetic diversity of the studied genomes.

### Single Nucleotide Polymorphisms (SNPs)

Exploration of a large number of polymorphisms within the genomes requires fast yet reliable, simple, and cost-effective techniques. Recently, SNP markers gained much interest and are a popular marker system of choice. SNP markers represent variation at a single DNA nucleotide site. Occurring throughout the genome in both protein-encoding and non-coding loci, SNPs are useful for a variety of population genetic and genomic applications. They can be detected and analyzed between individuals belonging to the same species (Mammadov et al., 2012). Typically, SNP frequencies are in a range of one SNP for every 100–300 bp in plants (Lateef, 2015). The strength of SNP relies on the large number of loci that can be assessed. In a low diversity species in which rare SNPs could be discovered, the power of discriminating the genetic population can be equivalent to the number of loci in a genetically diverse species (Foster et al., 2010).

Single nucleotide polymorphism-based studies in the Arabian Peninsula were emphasized on genetic diversity of barley cultivars, while few others investigated genetic variation from wheat, rice and palm (Figure 2). Moragues et al. (2010) evaluated single nucleotide polymorphic sites and selection strategies to estimate the germplasm diversity and population structure of different barley germplasms (landrace and cultivar), including the landraces of Jordan. All marker subsets gave qualitatively similar estimates of the population structure in both germplasm sets. Another study exhibited an innovative approach, where for the first time the oligonucleotide pool assay based on the SNP platform was used for assessing the evolution of barley varieties (landrace and wild) in a fertile crescent from Jordan. The study showed significant chromosome level variations between barley types, further suggesting hybridization and continued adaptation of landrace barley under cultivation (Russell et al., 2011). In addition, Hubner et al. (2012) investigated the extent of gene flow between wild barley *Hordeum spontaneum* and cultivated barley *Hordeum vulgare* in Jordan. The authors evaluated the effect population structure. The SNP markers revealed correspondence of the population structure to the major eco-geographic regions. Along with Jordan, plant samples from other countries like Oman, Iraq, Saudi Arabia, and Yemen were also involved in the SNP studies on barley. Xia et al. (2013) investigated SNP from the heat shock protein (HSP 17.8) gene across the barley population (*n* = 210), with samples collected from 30 countries. The population from Middle East Asian countries (including Iraq and Jordan) showed higher nucleotide diversity than the other regions. In addition, the wild-type population exhibited greater diversity than the cultivated population. Further studies might provide new insight in studying the potential genetic contribution to drought tolerance in barley (Xia et al., 2013).

Apart from barley, Zhang et al. (2012) studied mitochondrial and chloroplast genomes from two strains of Saudi Arabian Hassawi rice (*Oryza sativa*). They discovered new indels and SNPs in addition to a new type of sequence variation, termed as reverse complementary variation (RCV) as found in rice chloroplast genomes. Another study of Saudi Arabian palm cultivars on mitochondrial and plastid genomes also revealed plastid heteroplasmy, though low levels of variations were observed in both genomes (Sabir et al., 2014b). In Yemen, the association of North American spring wheat breeding mapped germplasm resistance against stem rust race using SNP markers (*n* = 27) (Bajgain et al., 2016). The authors found resistance for the highly virulent races, which could assist in the development of varieties with elevated levels of resistance. Another study on wheat (spring bread var.) exhibited thousands of new SNP variations in the landraces, which were well adapted to drought and heat stress environments (Sehgal et al., 2015).

As seen in the studies above, conventional molecular and genomic techniques are powerful during plant research, particularly in desert plants. The research findings can be utilized in biodiversity conservation programs. Additionally, reviewing a large body of case studies on desert plants would help evaluate the extent these markers and techniques can be useful for further studies.

## Advanced Techniques Associated With Plant Diversity Studies

In the last two decades, molecular markers revolutionized the study of plant science in the areas of genomics, transcriptomics, proteomics, metabolomics, etc., altogether emphasizing this approach as the science of “omics” (Mosa et al., 2017b). These techniques were mostly fueled by the emerging new technologies of next and third generation nucleic acid sequencing, as well as second-generation peptide sequencing platforms.

### Transcriptomics

Transcriptomics involves the study of transcripts which are formed from the complete set of RNA that are produced by the genome under specific circumstances or in a specific tissue. These transcripts can be detected using high-throughput methods, such as DNA microarray and RNA-Sequencing. The comparison of transcriptomes can facilitate identification of genes that are differentially expressed in distinct cell populations, or in response to different treatments (Mosa et al., 2017a). Thus, functional genetic diversity in plants can be effectively analyzed over the stress event, as in *Rhazya stricta*, an evergreen shrub from Saudi Arabia upon which the salt stress response was analyzed. Their results suggested high expression levels of the responsible genes [pentatricopeptide repeat (PPR) proteins] regulating large number of transcripts under salt stress (Hajrah et al., 2017). Transcriptomics also has the potential to reveal the rate of genome alteration. For instance, nucleotide substitution was reported among the intra-varietal SNPs in date palm (*Phoenix dactylifera*) cultivars (Khalas, Fahal, and Sukry) exhibiting slightly higher transversion rates than that of transitions. Apart from substitution, insertion of plastid DNA into the mitochondrial genome could result in size expansion, as seen in the date palm (*Phoenix dactylifera*) (Fang et al., 2012). The occurrence of duplication could also result in considerable variation, as seen in the transcriptome analysis of the previously discussed medicinal plant *R. stricta* in Saudi Arabia (Park et al., 2014). The competency of transcriptomics to reveal nucleic acid variation exhibits its ability to identify differentially expressed genes, events of genetic divergence and discrimination of various plant races.

### Proteomics

Proteomics is the analysis of all protein complement of an organism under a specific, defined set of conditions or in a biological system. In order to identify individual proteins, there has been considerable technological development through the past decades, with the separation technique being the most commonly used method in proteomics today (Yu et al., 2010). These developments include advances in mass spectrometry (MS) technology, protein fractionation techniques, and bioinformatics tools to analyze and assemble the MS data.

When proteins were compared among the species of specific plant races, they differed in exhibiting genetic variation, as observed in characterized protein patterns among all cultivars of *Heliotropium dignyum* (Alwhibi, 2017). The seed storage protein was characterized from different samples of the shrub *H. dignyum*, collected from different locations in Saudi Arabia. The results showed that the amounts of protein were different, despite being from the same geographical region. Leaf proteome analysis of the date palm (*Phoenix dactylifera*) was carried out to identify proteins involved in salt and drought stress tolerance. The analysis revealed differentially expressed genes demonstrating high or low protein abundance (El Rabey et al., 2016). Thus, proteomics could assist in differentiating gene contents due to functional variation occurring under environmental stress conditions.

### Metabolomics

Metabolomics is the study of all metabolites. Small molecules are the metabolic products generated by the process of metabolism in every cell and tissue, referred to as the metabolome of a biological system. Spectroscopy-based metabolic profiling technologies that can be applied to investigate the metabolic changes between different plant species and cultivars are mass spectrometry (MS) and nuclear magnetic resonance (NMR) (Mosa et al., 2017a). Plants display remarkable genetic plasticity and have developed an extraordinary range of genetically distinct and metabolically diverse cultivars for given plant species. Schauer et al. (2005) evaluated metabolic diversity in trait attributes of the non-domesticated *Solanum lycopersicum* species. The goal was to identify biochemical markers associated with a desired trait and then apply them for direct progeny selection when crossed with the domesticated ones. Using GC-MS methodology, the authors were able to generate profiles for a number of secondary metabolites, concluding that boosting levels of nutritionally important metabolites have a higher chance of success. In wild species, higher levels of secondary metabolites were observed, suggesting a valuable resource for flavor and color. Studies associated with stress tolerance in maize seedlings and roots have been reported to accumulate proline. On the other hand, drought conditions resulted in an increase in the levels of glycine betaine in maize leaf during growing seasons (Saneoka et al., 1995; Yang et al., 1995). Thus, susceptible plant species could withstand certain stress conditions. Metabolomics favors the assessment of genetic and phenotypic diversity of wild relatives of important crops, which may prove to be a valuable resource in eliciting new traits to the consumer and environment.

## Plant DNA Barcoding: An Emerging Approach

**FIGURE 3:**
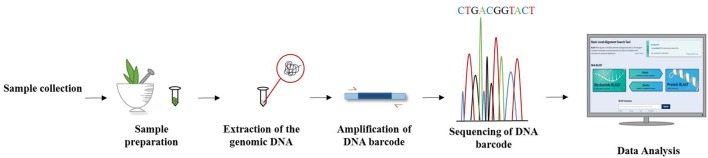
Workflow of DNA barcoding technique from specimen collection till barcode analysis.

DNA barcoding of plants has become an invaluable tool in taxonomic classification and the identification of species by sequencing a very short standardized DNA sequence in a well-defined gene (Figure 3). The success of this molecular and genomic technique in distinguishing species offers great hope for identifying unidentified specimens which might not be identified on morphological grounds alone. It is well understood that unlike animals, mitochondrial plant genes perform unsatisfactorily as a candidate sequence for DNA barcoding. Thus, DNA barcoding in plants has been considered a more challenging task than in animals. Ideally, a DNA barcode should allow unambiguous species identification by having sufficient sequence variation between closely related species. As there is no single universal barcode candidate for identifying all plant groups, a comparative analysis of plant barcode loci is essential for choosing the best candidates for authenticating particular plant genus/families (Awad et al., 2017). Researchers have advocated the use of two or more chloroplast barcodes for the best discrimination in estimating biodiversity, delimiting species and understanding species boundaries. The Plant Working Group (PWG) of the Consortium for the Barcode of Life (CBOL Plant Working Group et al., 2009) analyzed several chloroplast genomic regions across the plant kingdom and came up with standard plant DNA barcode combinations that increase the level of identification accuracy through incorporating both maturase K (*matK*), which offers high resolution and less universality, and ribulose 1,5-bisphosphate carboxylase/oxygenase large subunit (*rbcL*), which also offers high universality but less species resolution (Sathishkumar et al., 2015). By using chloroplast DNA barcodes, impressive progress has been made in identifying plant species (Kress and Erickson, 2007). Additionally, several chloroplast gene regions are typically used as plant barcodes along with *matK* and *rbcL*, which are considered as core barcodes (CBOL Plant Working Group et al., 2009). However, the power of this combination decreases when discriminating between closely related species. The addition of a nuclear internal transcribed spacer (*ITS*) to the chloroplast *matK* and *rbcL* combination as a supplementary marker can enhance the discrimination process (de Vere et al., 2012). Hence, there are some standard regions of DNA recommended for barcoding studies, including nuclear DNA (e.g., *ITS*) and chloroplast DNA (e.g., *rbcL, trnL-F, matK, psbA, trnH, psbK*) (Janzen et al., 2009; Vijayan and Tsou, 2010; Hollingsworth et al., 2011).

DNA barcoding is a useful tool for identifying species and plant parts nowadays as it neither requires specialized taxonomic knowledge nor information about the full length of the genome. In addition, it is not affected by morphological characteristics or by physiological conditions. This is possible due to the vast amount of taxonomically identified DNA barcodes being submitted at NCBI GenBank and Barcode of Life Datasystems. The taxonomic identification tool at Barcode of Life Datasystems facilitates identification of unknown species from their DNA sequences. Furthermore, this DNA-based examination technique can be implemented to appoint a taxonomic group for the query specimen to clarify the species boundary, build phylogenetic trees, and in biodiversity utilization and conservation of plant species by understanding evolution and ecology (Kress et al., 2015).

Traditional taxonomy based on morphological feature observation and chemical/biochemical methods misleads plant identification if applied on powdered or processed plant materials (Mosa et al., 2018). Moreover, experience in taxonomical examinations is necessary in order to reduce incorrectly drawn conclusions. An important point must be considered and highlighted while dealing with plants; their materials can undergo several physiological changes in storage conditions, which adversely affects the proper identification process (Mishra et al., 2016). However, the barcoding platform for analyzing plants at the genomic level could overcome the problems associated with correct identification while providing reliable results. As a means to revitalize traditional taxonomy, DNA barcoding has recently received increased recognition for the identification and delimitation of various plant species worldwide. Nowadays, authentication by DNA barcoding in plants can be easily obtained through raw herbs and definite amounts of finished dietary supplements in case of insufficient quantities of obtained DNA (Ramalingam et al., 2015).

Braukmann et al. (2017) utilized the most commonly used plant DNA barcodes (*rbcL, matK, and ITS2*) for a comprehensive analysis of 96% of the Canadian flora and reported that these markers were highly successful in identifying plants at the genus level (91–98%). The results indicated that the discriminatory power of these barcodes for the Canadian vascular plant species varies depending on the method of analysis and biogeographic region. The highest resolution was provided by *matK*, followed by *ITS2* and *rbcL*. However, the markers varied in their success of coverage across the species pool. Xu et al. (2018) demonstrated that DNA barcoding is an efficient tool for identifying invasive species, and *ITS/ITS2* + *matK* are the most suitable barcodes for invasive plants in China. As stated by Kress (2017), plant DNA barcoding will advance to serve the botanical community for building a more comprehensive plant DNA barcode library globally for universal use and developing new markers and adopting new sequencing for various forms of research. Among others, these studies have provided evidence that a plant DNA barcode with a high degree of utility can be implemented; they also illustrated problems that need to be overcome in order to achieve the ideal barcode.

The intervention of high throughput next generation sequencing (NGS) technologies now encompasses the Sanger sequencing platform in DNA barcoding (especially metabarcoding). In order to circumvent the current reliance of generating full length reference barcodes at a relatively slow rate using Sanger’s sequencing, NGS technologies would be highly efficient if used for DNA sequence acquisition. Furthermore, adoption of NGS technologies for DNA barcoding will lead to database expansion of the available sequence data and would be a powerful molecular tool for species discovery, evolution and the conservation of biodiversity (Sucher et al., 2012).

### Plant DNA Barcoding in the Arabian Peninsula

**FIGURE 4:**
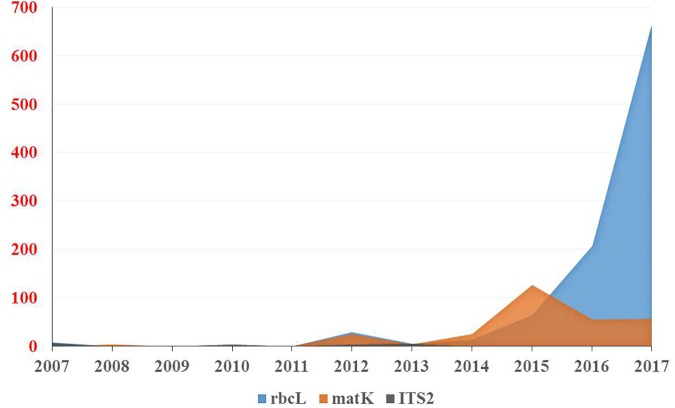
Number of DNA barcodes sequences submitted and published by NCBI GenBank in a decade from the Arabian Peninsula.

The results of our search analysis exhibit extensive use of the DNA barcoding technique over any other molecular and genomic methods for plant biodiversity assessment in the Arabian Peninsula (Figure 2B). To assess DNA Barcodes generated from the Arabian Peninsula in the last 10 years (2007–2017), we conducted a survey on NCBI GenBank to find the most sequenced marker reported from the Arabian Peninsula for the plant taxa. We mined core DNA barcode markers (*rbcL, matK* and *ITS2*) and found that the *rbcL* marker was the most sequenced barcode region, followed by *matK* and *ITS2* (Figure 4). Moreover, studies utilizing various DNA barcode markers to analyze genetic variation among plant species are listed in Table 1.

**Table 1:** Plant DNA barcode markers studied from the Arabian Peninsula.

Nation	Locus/Loci	Identification	Species barcoded (*n*)	Reference
Iraq	ITS4+ITS5	Cryptic medicinal plants	4	Mati and de Boer, 2011
Saudi Arabia	rpoB	*Phoenix dactylifera*	1 (8 cultivars)	Al-Qurainy et al., 2011b
	psbA-trnH			
Saudi Arabia	ITS1+ITS2	*Ruta graveolens*	1	Al-Qurainy et al., 2011c
Saudi Arabia	rbcL	Arid plants	26	Bafeel et al., 2011
	matK			
Saudi Arabia	rbcL	Arid plants	12	Bafeel et al., 2012b
				
Saudi Arabia	rbcL	Herbaceous weed (*Chenopodium murale*)	1	Bafeel et al., 2012a
	matK			
Saudi Arabia	ITS2	Medicinal plant (*Ochradenus arabicus*)	1	Khan et al., 2012
	rbcL			
	rpoB			
	rpoC1			
Saudi Arabia	ITS1+ITS2	Rare and endangered plants	5	Al-Qurainy et al., 2013
Saudi Arabia	ITS	*Reseda pentagyna*	1	Ali et al., 2013
	trnL-F			
Saudi Arabia	ITS1 + ITS4	*Senecio asirensis*	1	Khan et al., 2013
	rpoB			
	rpoC1			
	psbA-trnH			
	rps-16			
Saudi Arabia	rbcL	*Diospyros mespiliformis*	1	Alaklabi et al., 2014
Saudi Arabia	ITS1 + ITS2	*Nepeta deflersiana*	1	Al-Qurainy et al., 2014a
	rbcL			
	rpoB			
	rpoC1			
	rps16			
	psbA-trnH			
Saudi Arabia	ITS1 + ITS2	*Plectranthus asirensi*	1	Al-Qurainy et al., 2014b
	rbcL			
	rpoB			
	rpoC1			
	rps16			
Saudi Arabia	ITS1 + ITS2	*Echinops mandavillei*	1	Al-Hemaid et al., 2014
United Arab Emirates	matk	*Phoenix dactylifera*	1	Enan and Ahamed, 2014
	rpoC1			
Saudi Arabia	ITS1+ITS2	*Euphorbia scordifolia*	1	Al-Hemaid et al., 2015
United Arab Emirates	psbK-psbI	*Phoenix dactylifera*	1	Enan and Ahamed, 2016
United Arab Emirates	matk	Selected medicinal plants	10	Enan et al., 2017
	rbcL			
	rpoC1			
United Arab Emirates	rbcL	Native plants	51	Maloukh et al., 2017
	matK			


Our present work emphasizes studies undertaken from the Arabian Peninsula that have demonstrated the efficiency of DNA barcode markers in species identification and the assessment of genetic variation in different geographical regions (Table 1). Several studies for the authentication of medicinal herbs from the herbal drug markets in Saudi Arabia have been reported. The herb *Ruta graveolens* is morphologically similar to *Euphorbia dracunculoides*, which can be used as an adulterant. Their taxonomic authentication was done using *rpoB*, *rpoC1*, and *nrDNA-ITS* (Al-Qurainy et al., 2011b,c). In another study, a molecular signature of the economically important date palm (*Phoenix dactylifera*) by Al-Qurainy et al. (2011a) was able to differentiate among different cultivars by using the *rpoB* and *psbA-trnH* genes. The authors showed that *psbA-trnH* had more polymorphic sites than the *ropB*, and locus. Taxonomic evaluation is not the only application of DNA barcoding, as geographical variation was also studied by Bafeel et al. (2011) in plants from the arid environment of Saudi Arabia using *matK* and *rbcL* universal primers. They concluded a failure in certain cases of amplification due to primer mismatch at the annealing site. However, *rbcL* and *matK* can still be used for plant barcoding while the search for other primers with a broader coverage of plant species is still ongoing (Bafeel et al., 2011). However, researchers have argued that it is very difficult to find a universal barcode for the identification of all plant species due to morphological and geographical variations as well as reticulate evolution. In the Arabian Gulf region, plant species are able to withstand extreme and harsh conditions like salinity, drought, solar radiation and high temperatures in comparison to plant species in other parts of the world. Bafeel et al. (2012b) once again evaluated the potential of the *rbcL* marker for use in the identification of wild plants in these arid regions. They concluded that *rbcL* sequences identified 92% of the samples at the genus level but only 17% at the species level. In another study, Bafeel et al. (2012a) performed molecular characterization of *Chenopodium murale*, an invasive herbaceous weed species in Saudi Arabia with negative allelopathic effects to enhance the morphological identification system. Amplification of the barcoding genes *rbcL* and *matK* in *C. murale’s* plastid region indicated that *matK* possesses high discrimination efficiency and the lowest average pairwise sequence similarity in comparison to *rbcL*, as well as the fact that the combination of *rbcL* and *matK* could yield high resolutions. Another team from Saudi Arabia tried to identify and discriminate between some species of the genus *Ochradenus* viz., *O. arabicus* and *O. baccatus*, which are closely related and confusing species. They used universal primers (nrITS, *rbcL*, rpoC1, rpoB, and rps16) for the amplification of the nuclear ribosomal (nrDNA) and chloroplast (cpDNA) spacer sequences. Their results revealed that certain markers (*nrITS*, *rpoB* and *rpoC1*) were more informative by having larger sequence variations than the rest of the markers (Khan et al., 2012). Furthermore, Khan et al. (2013) used this tool to identify an endemic plant species, *Senecio asirensis*, that has some poisonous or non-poisonous components with medicinal value. They used *ITS*, *rpoB*, *rpoC1*, *psbA-trn* and *rps-16* for the identification and successfully located ITS, psbA-trn and rps-16.

Another endemic species, *Reseda pentagyna* (Resedaceae), was analyzed by Ali et al. (2013) using *ITS* of nrDNA and *trn*L-F of chloroplast sequences. They were able to distinguish *R. pentagyna* from the closely related species *R. stenostachya*, which differs only in the number of toothed capsules. Results revealed the proximity and maximal identity of Resedae with 100% bootstrap support and nested within the clade. Al-Qurainy et al. (2013, 2014a,b) analyzed five species of rare and endangered plants from Saudi Arabia. Al-Qurainy et al. (2014a,b) evaluated medicinal plants *Nepeta deflersiana* and *Plectranthus asirensi* using DNA barcoding. In their study, they used various markers (Table 1), among which ITS was suggested for rare and endangered plant species, including *P. asirensi* (with the exception of *N. deflersiana*). These species were successfully distinguished by using *rps16* and *psbA-trnH* barcode markers. For *Plectranthus asirensi*, the authors suggested *rbcL* and *rpoC1* as successful candidate barcode markers for its identification. Al-Hemaid et al. (2014,^ 2015^) studied the efficiency of *ITS* to resolve *Echinops mandavillei* and *Euphorbia scordifolia* respectively using nrITS (*ITS1* and *ITS2*). They used BLAST and phylogenetic analysis to distinguish these species. The results concluded that *ITS* has sufficient potential to resolve these taxa. Another deciduous medicinal plant, *Diospyros mespiliformis*, which is also endangered in Saudi Arabia, was studied for its plastid *rbcL* gene. The phylogenetic analysis succeeded in discriminating it from the other species with nucleotide variations in three different sites. Therefore, the *rbcL* short sequence region (664 bp) was preferred as a DNA marker for authenticating and identifying samples of *D. mespiliformis* (Alaklabi et al., 2014).

**FIGURE 5:**
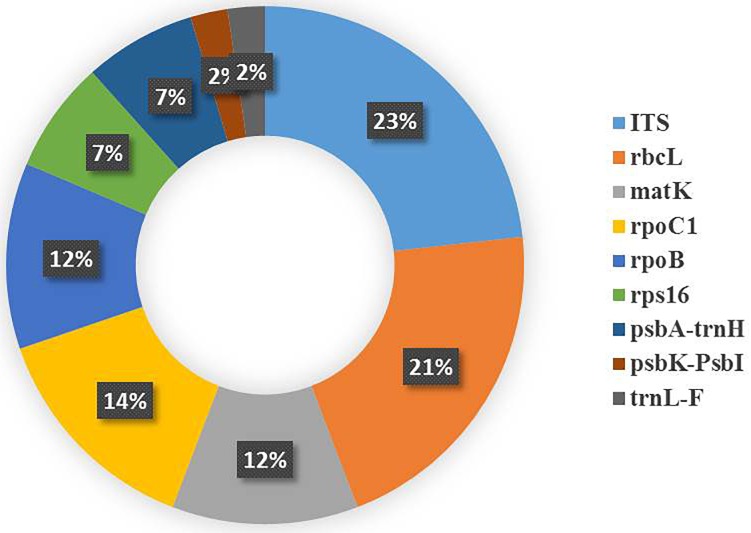
Pie chart representing the studies reporting the efficiency of the plant DNA barcode markers.

Recently, a few studies have been reported from the UAE and Iraq. The UAE was involved in authenticating date palm cultivars and a few medicinal plants (Enan and Ahamed, 2014; Enan and Ahamed, 2016; Enan et al., 2017). The authors analyzed *matK* and *rpoC1* barcodes in 11 different date cultivars, with *matK* deemed to be more useful (Enan and Ahamed, 2014). In addition, *psbK-psbL* was found as an eligible barcode marker in resolving the date palm taxon (Enan and Ahamed, 2016). In medicinal plants, the rate of PCR amplification for the desert plants collected freshly and from herbarium using *matK*, *rbcL* and *rpoC1* was also tested (Enan et al., 2017). The fresh samples showed amplification of 90%, 90%, and 80% at *matK*, *rbcL*, and *rpoC1* locus, respectively, which was better than the herbarium samples. The authors also concluded that the *rbcL* region can be considered as a potential marker to be utilized for distinguishing medicinal plants (Enan et al., 2017). In Iraq, a study reported the use of DNA barcoding to facilitate ethnobotanical trade in herbal samples (Mati and de Boer, 2011). About 82 species were collected, of which 4 cryptic taxa of medicinal plants were subjected to DNA barcoding using *ITS* markers. They were able to identify these plants by conducting NCBI BLAST. Overall, the DNA Barcode markers (including core and supplementary markers) that were utilized in the above studies were inferred from Table 1 and a pie chart was constructed to show the efficiency of the DNA barcode markers studied over a decade in the Arabian Peninsula (Figure 5).

## Role of Herbarium Collections in Plant Diversity Analysis

Herbaria are widely used as a resource for identifying plants, establishing their geographic range, describing new species and to provide an overview of the floral diversity of a region. According to recent estimates, herbaria contain about 480 million specimens worldwide, accumulated through the efforts of thousands of botanists/taxonomists (Thiers, 2017). Plant specimens held in herbaria and herbarium-type materials are crucial for accurate plant identification and taxonomy, comparative purposes in taxonomic or phylogenetic studies (Besnard et al., 2014), as foundations for the flora of a region, and to provide DNA samples for research on various aspects of plant biology. Researchers have considered them as potentially an enormous resource of materials in molecular phylogeny studies, including the construction of a DNA barcode library for the flora of a region (Délye et al., 2013; Xu et al., 2015). Several herbaria in the Arabia Peninsula exchange herbarium samples of different species. Having samples of certain species from different countries could help in defining the most genetically diverse population for conservation.

Dried plant herbarium specimens have been considered as a potentially valuable source of DNA (Staats et al., 2011). However, its extraction at a suitable quality for detailed molecular studies is often challenging because of frequent damage to DNA during storage (Gansauge and Meyer, 2013). It is also highlighted that the process of extracting DNA from herbarium specimens is often fraught with difficulty related to such variables as plant chemistry, specimen drying methods, and the chemical treatment of specimens (Drábková, 2014). Thus far, many methods have been developed for the extraction of DNA from herbarium specimens, with the most frequently used being either the traditional CTAB protocol (Doyle and Dickson, 1987), sometimes with modifications (Allen et al., 2006; Cota-Sánchez et al., 2006; Tarieiev et al., 2011) or DNA extraction kits such as the DNeasy Plant Mini Kit (Qiagen) (Drábková, 2014). Särkinen et al. (2012) outlined the major challenges of molecular studies using herbarium DNA and emphasized that despite the large number of specimens housed in herbaria worldwide, currently only a small fraction is being used for DNA-based research, mainly due to the poor success and difficulties in obtaining amplifiable DNA. The authors proposed that more systematic studies are needed to optimize methods and their efficiency in obtaining good quality DNA from herbarium specimens for the success of a molecular study. In a comprehensive study, de Vere et al. (2012) created a DNA barcode database for native and archaeophyte flowering plants and conifers in the nation of Wales. This represented the largest DNA barcode dataset to utilize herbarium material. However, there is no universal barcode approach for plants; researchers are using a number of different molecular markers for improving success in DNA barcoding.

In recent years, the number of phylogenetic studies using herbarium specimens has been gaining momentum. Researchers have been using herbarium specimens in molecular phylogeny studies and consider them as an interesting potential source of material for DNA barcoding and the construction of a barcode library for flora (Cozzolino et al., 2007; Erkens et al., 2008; Tarieiev et al., 2011; de Vere et al., 2012; Särkinen et al., 2012; Hebert et al., 2013; Xu et al., 2015; Bakker, 2017). In such studies, collections of herbaria are used when species are not easily collected in the field, since it is generally difficult to obtain living material of certain rare species (Délye et al., 2013). Additionally, the quality of DNA obtained from herbarium specimens should ideally be consistent with freshly collected samples for barcoding studies. Some researchers have successfully amplified DNA from herbarium specimens by modifying methods of DNA extraction (Cota-Sánchez et al., 2006; Andreasen et al., 2009; Staats et al., 2011; Tarieiev et al., 2011; Gutaker et al., 2017). However, some researchers have highlighted that it is challenging to find a suitable genomic region for DNA barcoding in herbarium samples in a wide range of taxa (Lahaye et al., 2008a,b; Poczai et al., 2009; Drábková, 2014). In such situations, NGS can allow the simultaneous generation of a large quantity of sequences for different genomes present in an organism (Glenn, 2011; Harrison and Kidner, 2011). Thus, advances in sequencing technology are providing new cost-effective options for genome comparisons on a much larger scale (Nock et al., 2011). The floristic and genetic information housed in herbaria provide an exciting prospect to use tissue samples from herbarium specimens as a source of sequence data for DNA barcoding, and their applicability for genetic, ecological, and environmental studies (e.g., Pyke and Ehrlich, 2010; Staats et al., 2011; Culley, 2013).

In instances where obtaining a representative sample of plant species from diverse areas such as the Arabian Peninsula can be a logistical challenge, the use of already collected herbarium samples can potentially overcome such barriers in many phylogenetic analyses. Furthermore, in cases where no living plant material exists or is not available for various experiments, DNA can sometimes be obtained from historical and modern herbarium specimens despite the potential for degradation due to age and storage conditions (e.g., Lister et al., 2010; Zuntini et al., 2013). It has also been highlighted that current DNA extraction methods involve destructive sampling of the specimen (e.g., removal of a leaf and subsequent grinding), which can limit the future use of a specimen for botanical research (Shepherd, 2017). Thus, it is desirable to use DNA extraction methods which would minimize damage to specimens. Ideally, the reference collections in herbaria, botanical gardens, museums and other repositories are critical resources for research, education, and helping the conservation of biological diversity. Despite the large number of already existing specimens in herbaria worldwide, only a small fraction is currently being used for DNA-based research. Hence, more systematic studies need to be conducted to optimize methods and their efficiency (Särkinen et al., 2012).

**FIGURE 6:**
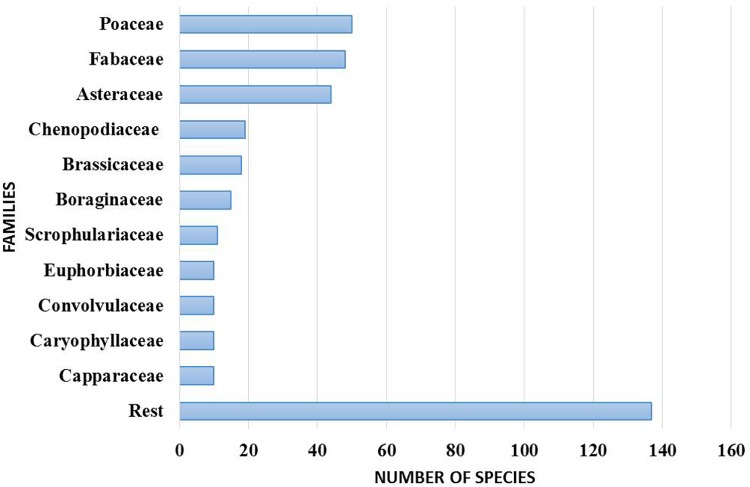
Number of species in dominant families in the Sharjah Seed bank and Herbarium (SSBH).

The Sharjah Seed Bank and Herbarium (SSBH) provide an important source of reference for the UAE flora (Figure 6). The herbarium provides opportunities for undertaking biodiversity research on the UAE flora in the widest sense, including plant anatomical and morphological analysis, taxonomy, classification and phylogenetic studies. Concerning DNA barcoding using herbarium specimens, plant specimens in the SSBH can be an invaluable resource in constructing a DNA barcode library of the UAE’s flora. The herbarium can provide taxonomic identification and survey services to enable more precise identification of plant samples which are difficult to discriminate morphologically. Literature suggests that the UAE’s native plant flora is under-explored in terms of detailed taxonomic and molecular phylogenetic studies. At present, the herbarium contains more than 4000 specimens of vascular plants, and new specimens are continually added as a result of extensive fieldwork in different parts of the country. These specimens belong to 382 identified species from 254 genera and 65 families.

## Conclusion and Future Prospects

Molecular and genomic techniques have always been indispensable tools for taxonomic identification and genetic diversity analysis. Traditional techniques include RAPD, RFLP, AFLP, ISSR, SNP, Microsatellites, ESTs and SCAR, which are undoubtedly valuable tools in understanding the molecular diversity between closely related species as well as the population genetics of species encompassing a wide range of habitats. In addition, such techniques could help in generating baseline data for undertaking plant breeding programs. These markers have their own potential in addressing the above issues but possess some restrictions in relation to phylogeny reconstruction and taxonomy, possibly due to lack of universality or ideal markers for species recognition, which could pose problems or even mislead. Being a fast-moving field with the advent of new techniques, DNA barcoding has led toward its goal to resolve taxonomic issues through phylogenetic reconstruction by recognizing the mitochondrial COI gene as a universal barcode marker. Unfortunately, the universality of this gene has been restricted to animal taxa. For plants, chloroplast markers *rbcL* and *matK* have been recognized as core barcode markers, in some cases accompanied with supplementary nuclear markers. Recent technologies have also been upgraded to enable the acquisition of huge amounts of data through high throughput sequencing, termed as NGS techniques. With the advent of NGS technologies, extensive studies are being undertaken based on cDNA array-based gene expression, identification of various patterns (microsatellites) in genes of different organisms, and their metabolic pathways. Moreover, this technology efficiently generates high quality DNA barcodes that could improve species diagnostic power, which appears to be promising (Wilkinson et al., 2017). Additionally, the integration of multi-omics technologies such as transcriptomics, proteomics, and metabolomics would enable researchers to deeply understand the genetic diversity of the Arabian Peninsula flora.

Arabian Peninsula countries have arid/hyper-arid subtropical climates. However, there is great heterogeneity in the environmental conditions. Interestingly, many of the species overlap in most of these countries. It is plausible to use different genetic markers to assess the genetic diversity among the economically important and endangered species within the whole range of the Peninsula. Such a study would help in defining the most genetically diverse population that could be either used for economically important species or conserved for critically endangered species. For example, seed dormancy and germination habitat requirements in different halophytes (i.e., grow well in both saline and non-saline habitats) have been studied (El-Keblawy et al., 2018). The authors reported significant differences in germination requirements and dormancy among seeds of saline and non-saline habitats. They attributed such differences to transgenerational induction, which is cued by an environmental signal in the parental generation and is expressed independently of changes in the offspring genotype. Transgenerational mechanisms can occur through maternal and/or epigenetic effects (El-Keblawy et al., 2018). It is important to define the mechanisms behind the differences between plants of the two habitats (Donohue, 2009; Soliman et al., 2018).

In addition, global warming can affect plant distribution and may cause extinction, especially in mountains which harbor a unique and large portion of the world’s biodiversity (El-Keblawy, 2014). Defining more biodiversity-rich populations and the proper habitat types for conserving endangered and endemic species (especially those at the top of mountains) should be among the top research priorities in the area. Very few studies have assessed the genetic diversity of endemic plants in the Arabian Peninsula (e.g., Ali et al., 2013; Al-Qurainy et al., 2014a). However, no study has yet assessed genetic diversity in different ranges of species distribution.

In order to maintain taxonomic reliability of morphologically identified specimens, it is important that they be accompanied by DNA barcodes. As such, herbarium specimens along with DNA barcodes would provide a trustworthy archive for identifying native plants and their ecological expanse. The herbarium at SSBH provides opportunities for undertaking biodiversity research on the UAE flora in the widest sense. There is a scarcity of studies emphasizing the application of DNA barcoding in taxonomic identification and the generation of a barcode library for plants in the Arabian Peninsula, specifically the UAE. We conclude with our proposition in the area of DNA barcoding through the establishment of a DNA barcode toolbox using herbarium specimens in the SSBH, which possess accurately identified voucher specimens assigned to barcode sequences. Thus, this invaluable resource would assist non-taxonomists and policy makers to promote future resource monitoring programs and decide conservation strategies for plants in the UAE.

## Author Contributions

KM conceived the idea, designed the outlines, conceptualize the overall structure, and edited the manuscript. KM, SG, KA, EA, and AE-K drafted the manuscript and prepared the illustrations. RJ analyzed the data, prepared the figures, and edited the manuscript. KM, SG, and AE-K critically edited the manuscript. HS and TM helped on literature review.

## Conflict of Interest Statement

The authors declare that the research was conducted in the absence of any commercial or financial relationships that could be construed as a potential conflict of interest.
